# Conditional survival estimates for cancer patients

**DOI:** 10.18632/oncotarget.21497

**Published:** 2017-10-04

**Authors:** Lauren E. Haydu, Richard A. Scolyer, John F. Thompson

**Affiliations:** John F. Thompson: Melanoma Institute Australia, The University of Sydney, and Sydney Medical School, The University of Sydney, Sydney, NSW, Australia; Royal Prince Alfred Hospital, Camperdown, NSW, Australia

**Keywords:** conditional survival, cancer, prognosis, staging

For patients with cancer, the first and most important assessment of prognosis occurs at the time of initial presentation. This prognostic estimate is based on detailed assessment of clinical, pathologic and imaging information. The recently-published Eighth Edition of the American Joint Committee on Cancer (AJCC) Staging Manual [1] provides a robust contemporary, disease-specific estimate of prognosis at initial cancer presentation. The AJCC stage groupings guide clinical decision-making and determine clinical trial eligibility and stratification. The mammoth effort of the AJCC in producing its new staging manual brings us a step closer to personalized cancer prognostic assessment, and more nuanced integration of detailed disease features into an increasingly complex staging system will inevitably evolve into individualized prognostic calculators in future iterations of the AJCC Staging Manual.

Following initial presentation, it becomes important for both the patient and their attending clinician to know how prognosis will change over time on the *condition* of the patient surviving. Estimates of survival based on assessment at initial presentation, such as those used by the AJCC, will be heavily weighted by early deaths from disease and can mislead surviving patients, for whom long-term prognosis may actually improve substantially as their survival time extends. Conditional survival estimates provide a dynamic assessment of long-term outcomes for patients who survive their cancer for given periods of time. The factors that stratified prognosis at the time of initial presentation may not determine longer-term risk profiles, and an understanding of the relevant factors that determine prognosis at subsequent time-points is likely to identify a group of patients requiring more intensive long-term follow-up, for whom significant risk factors persist years after diagnosis. Conversely, it may also be possible to reduce the intensity of follow-up for some patients, thereby reducing the resource burden on health systems. As well, the anxiety of other patients who may actually now have a substantially lowered risk of death, even approaching that of the general population, may be alleviated. This refinement of the science of prognostic assessment should thus serve those at both ends of the disease spectrum; those at high to moderate risk, likely to benefit from adjuvant therapies and frequent routine follow-up, and those at low risk, who can move on with their lives with reasonable confidence that disease recurrence is unlikely to occur.

In a study by our group, published recently in the *Journal of Clinical Oncology* [2], conditional survival was assessed for a large cohort of melanoma patients (n=4540) with loco-regionally metastatic disease (Stage III), with adjustment for age, sex and AJCC 8^th^ edition stage group (IIIA, IIIB, IIIC, IIID). With clinical trials of new neo-adjuvant and adjuvant treatment strategies now in progress for this group of melanoma patients, it is important to understand their dynamic, long-term prognosis, in an individualized context. Analysis of our cohort revealed that males, older patients, and those with a higher Stage III subgroup were at highest risk of death, particularly within the first 2 years after diagnosis. However, after three years of survival, 5-year survival estimates for male and female patients were no longer significantly different. Similarly, 5-year survival outcomes for patients who presented with Stage IIIA and Stage IIIB disease also converged after surviving three years.

A similar analytic approach was used by Kim et al to investigate conditional overall survival (OS) and disease-free survival (DFS) outcomes for 723 patients with non-small cell lung cancer (NSCLC) [3].In their article, published this year in *Oncotarget*, conditional 3-year OS and DFS estimates at baseline and at 1, 2, 3, 4 and 5 years after initial presentation were reported. A comprehensive range of prognostic factors was assessed. The authors appropriately commented in their discussion that the frequency of patient follow-up is often tapered despite limited evidence to support that decision. In fact, the results of their study indicate that longer and/or more intensive surveillance of some patients, notably those with either a high preoperative tumor SUVmax on PET-CT, or a low tumor-shadow disappearance ratio on CT, may be warranted.

In an analysis of 615 patients with advanced laryngeal cancer, published in *Nature Scientific Reports* this year [4], the Multidisciplinary Larynx Cancer Working Group reported conditional survival estimates for the first time for this disease type. The authors found that longer-term outcomes following initial management can be affected by treatment-related toxicity and continued alcohol and tobacco use, causing comorbidities unique to patients with laryngeal cancer. Six-year overall survival for the entire cohort was 49% (95% CI: 43-53%). This was significantly worse than the three-year survival of patients who had already survived three years (72% [95% CI: 65-74%]). Conditional survival analysis of additional patient factors also revealed significant differences in long-term outcomes according to race, providing evidence for increased targeted surveillance of black patients.

In each of these three important conditional survival studies, it was possible to identify a group of patients with an increased risk of recurrence and death, likely to benefit from more intensive follow-up after initial disease management. Conversely, dynamic conditional prognostic assessments identified other patients with *improved* long-term outcomes, for whom frequent, costly and anxiety-provoking follow-up visits and investigations may not be warranted, and who may be reassured that the likelihood of disease recurrence and death is low. Conditional survival estimates for cancer patients will not only inform treatment and follow-up, but will also provide surviving patients with more accurate knowledge of what their future is likely to hold.
